# Vascular Complications in Transcatheter Aortic Valve Replacement Using 14 vs. 18 French Plug-Based Percutaneous Closure Devices: A Propensity Score-Matched Observational Study

**DOI:** 10.3390/jcm15083095

**Published:** 2026-04-18

**Authors:** Tobias Lerchner, Norvydas Zapustas, Melchior Seyfarth, Klaus Tiroch, David Holzhey, Marc Michael Vorpahl

**Affiliations:** 1 Doctoral Programs, Witten-Herdecke University, 58455 Witten, Germany; 2 Department of Cardiology, Helios University Heart Center, Witten-Herdecke University, 42117 Wuppertal, Germany; 3 Department of Cardiology, Heart Center Bodensee, Witten-Herdecke University, 78464 Konstanz, Germany; 4 Department of Cardiothoracic Surgery, Helios University Heart Center, Witten-Herdecke University, 42117 Wuppertal, Germany; 5 Department of Cardiology, Helios Heart Center, Witten-Herdecke University, 53721 Siegburg, Germany

**Keywords:** plug-based vascular closure, TAVR, structural heart, access site complications, 14 French

## Abstract

**Background/Objectives:** Plug-based vascular closure devices (Pb-VCDs) are routinely used in 14 and 18 French (F) size for percutaneous vascular access site closure during transfemoral transcatheter aortic valve replacement (TAVR). Recently, larger 18F Pb-VCDs were linked to increased incidence of vascular complications in randomized comparisons. Smaller 14F devices are hypothesized to decrease the incidence of vascular complications, but real-world data on their safety in routine clinical practice is scarce. **Methods:** We performed a retrospective, propensity score-matched comparison of patients receiving either 14F or 18F Pb-VCDs during TAVR from March 2019 to December 2020. The choice of 14F or 18F Pb-VCD utilization depended on the sheath size during the procedure. No other vascular closure systems (VCDs) were used despite the MANTA (Teleflex Inc.^®^, Morrisville, NC, USA) Pb-VCD. The primary endpoints were major and minor vascular complications defined by valve academic research consortium-3 (VARC 3) criteria. Secondary endpoints included VARC-3 bleeding events, length of hospital stay and in-hospital mortality. **Results:** A total of 183 (14F Pb-VCD) and 110 (18F Pb-VCD) patients were included in 1:1 propensity score matching and resulted in 85 matched patient pairs. The primary endpoint of major and minor vascular complications was balanced between the groups (major: 3.5% (14F Pb-VCD) versus (vs.) 0.0% (18F Pb-VCD), *p* = 0.25; minor: 12.9% vs. 14.1, *p* = 1.00). Secondary endpoints of VARC-3 bleeding events (*p* = 1.00), length of hospital stay (*p* = 0.34), and in-hospital mortality (*p* = 1.00) were equally distributed. **Conclusions:** There is no difference in major and minor VARC-3-defined vascular complications between the 14F and 18F groups in our study. Following this real-world observational analysis, observed rates of vascular complications need to be validated in prospective controlled trials.

## 1. Introduction

Aortic valve stenosis (AS) is the most common valvular pathology in Europe and patients affected present with various phenotypes including mild, moderate or severe manifestations [1,2]. AS is defined as severe if an aortic valve area (AVA) of ≤1 cm^2^ (indexed AVA ≤ 0.6 cm^2^/m^2^), peak aortic velocity ≥ 4 m/s and mean gradient ≥ 40 mmHg are documented during echocardiography [3].

Once patients develop severe, symptomatic AS, two different treatments are available: surgical aortic valve replacement (SAVR) and interventional implantation of a new aortic valve using a catheter system (transcatheter aortic valve replacement; TAVR). Here, the gold standard of treatment is an access by puncturing the common femoral artery (CFA) followed by transfemoral TAVR implantation (TF-TAVR) [4].

To adequately close the femoral access site after successful implantation of the valve system, percutaneous vascular closure devices (VCDs) are used [5]. VCDs can withstand high arterial pressures and are therefore a suitable tool to ensure safe and effective closure of the femoral vessels by the means of hemostasis [6]. Two types of VCDs are mainly in the use for TAVR access closure: suture-based (Sb-VCD) and plug-based VCDs (Pb-VCD) [7].

The MANTA (Teleflex Inc.^®^, Morrisville, NC, USA) Pb-VCD belongs to a new generation of VCDs used for closure of large bore arteriotomies. Since 2016, MANTA (Teleflex Inc.^®^, Morrisville, NC, USA) is a CE-certified alternative to the established suture-based systems [8] and an increasing number of dedicated heart centers use Pb-VCDs as a primary percutaneous closure technique or first-line alternative to Sb-VCDs during TF-TAVR [9]. Pb-VCDs are used in 14 French (F) and 18F sizes in daily practice, but recently, clinical trials focused on larger 18F Pb-VCDs only, which were associated with increased rates of vascular access site complications in comparison to Sb-VCDs [5]. Despite being not interchangeable, there is only little evidence regarding the impact of 14F Pb-VCDs on the rates of vascular complications during TAVR and the sparse number of trials regarding 14F sized Pb-VCDs available were conducted during early MANTA (Teleflex Inc.^®^, Morrisville, NC, USA) days [10], included only a small cohort [11], or used outdated valve academic research consortium-2 (VARC-2) criteria [7].

Although sheath selection follows standardized thresholds, the transition from a 14F to an 18F-VCD represents a relevant step-up in arteriotomy closure strategy, potentially associated with increased vessel trauma, larger collagen plug volume, and different biomechanical interactions with the femoral artery. Whether this translates into clinically meaningful differences in vascular outcomes has not been sufficiently addressed in prior studies. Given their smaller device profile and reduced arteriotomy size, 14F Pb-VCDs are expected to be associated with lower rates of vascular access site complications compared with 18F systems. In this context, patients with smaller or more fragile femoral vessels, relevant peripheral artery disease, or an increased bleeding risk may particularly benefit from the use of lower-profile (14F) devices. In contrast, larger-bore devices (18F) may be required in procedures necessitating larger sheath sizes and may be more suitable in patients with adequate vessel caliber and favorable vascular anatomy. Thus, device selection should be guided by both procedural requirements and individual patient characteristics.

In this study, we aim to evaluate the impact of a smaller 14F size for percutaneous access site closure during TAVR has on patients’ safety. Therefore, we investigated the incidence of major and minor valve academic research consortium-3 (VARC-3)-defined vascular complications, VARC-3 bleeding events, in-hospital mortality and length of hospital stay after 14F and 18F Pb-VCD use in a single-center trial with patients undergoing TF-TAVR.

## 2. Materials and Methods

### 2.1. Study Design and Population

Our study is a clinical retrospective, propensity score-matched study evaluating the rates of vascular complications after Pb-VCD use during TF-TAVR at the Heart Center of the Helios University Hospital Wuppertal. The study was approved by the local ethics committee of the Heart Center of the Helios University Wuppertal/University Witten-Herdecke (114/2021) and all work involving human subjects was conducted in accordance with the World Medical Association Declaration of Helsinki. Further, all research was performed in accordance with relevant guidelines and regulations, and informed consent was obtained from all participants and/or their legal guardians.

A total of 293 consecutive patients received the MANTA-VCD (Teleflex Inc.^®^, Morrisville, NC, USA) as only VCD for access site closure during TF-TAVR in 2019 and 2020 and were included in propensity score matching. No other VCDs despite the plug-based MANTA (Teleflex Inc.^®^, Morrisville, NC, USA) device were used in our center during this study. A total of 183 patients were assigned to the 14F and 110 patients to the 18F group before matching was performed. Propensity score matching resulted in 85 patient pairs. Exclusion criteria for this study included utilization of a Sb-VCD or a Pb-VCD other than the MANTA (Teleflex Inc.^®^, Morrisville, NC, USA) device, and an access site different to the transfemoral route, i.e., transapical or subclavian access or surgical repair for vascular complications. No additional criteria for exclusion were applied, and consecutive patients were retrospectively included in this study.

### 2.2. Pre-Interventional Assessment and Planning of Peripheral Vascular Route

Pre-interventional examinations were used to assess aortic stenosis and to plan TF-TAVR using standardized procedures and common examination methods. For evaluation of the vascular access, computer tomographic images were screened for the minimal diameter of the CFA, the location of the femoral artery bifurcation and the presence and severity of tortuosity of the ilio-femoral vessels [12]. Calcifications at the CFA were classified as mild, moderate, or severe if present [13]. For precise implantation of the TAVR heart valves and assessment of peripheral access site anatomy, the Siemens Healthineers GmbH© syngo.via system (Munich, Germany) was used. Vascular tortuosity was assessed using contrast-enhanced computed tomography. Recommended cut-off values were utilized (mild (30–60°), moderate (60–90°), or severe (≥90°)) as previously described [12,13]. Grading of vascular tortuosity was discussed in a dedicated heart-team, as recommended by European guidelines [14].

### 2.3. Pb-VCD During TAVR Implantation

In this patient population (n = 293), the MANTA (Teleflex Inc.^®^, Morrisville, NC, USA) system was the only VCD used during TAVR. The choice of 14F or 18F Pb-VCD utilization depended on the provided instructions for use of the MANTA Pb-VCD (Teleflex Inc.^®^, Morrisville, NC, USA). Thus, the 14F system was used for the closure of femoral arterial access sites following the use of 10–14F devices or sheaths, whereas the 18F Pb-VCD was used for the closure of femoral access sites following the use of 15–20F devices or sheaths. TAVR was performed by three experienced operators (>500 TAVR as a first operator). Details on how the system was used were described previously [5,15,16]. In brief, the VCD was advanced percutaneously and consisted of an 8F dilator to expand the puncture site, a dedicated sheath, an occlusion unit, and a delivery system. It was used in sizes 14F and 18F to close arteriotomies of 12F–25F. Before starting TAVR, the depth of the femoral puncture site was determined. For this purpose, the Pb-VCD sheath was advanced into the vessel lumen at the access site and continuously retracted. The numbering on the side edge of the Pb-VCD was used to determine the depth of penetration based on the blood flowing back from the Pb-VCD. The dislocation depth resulted from the determined penetration depth plus 1 cm. After implantation of the TAVR heart valve, the sheath was removed and replaced with the Pb-VCD sheath via a stiff guide wire. This was fully inserted into the vessel. The introducer was then removed and the Pb-VCD was connected to the sheath and inserted into the vessel. The Pb-VCD was retracted through a 45° angle to the previously determined deployment depth. The rotation of the dislocation lever released the intravascular anchor made of a polymer of polylactide-co-glycolide (PLGA). As the Pb-VCD was further withdrawn, the anchor was pressed intravascularly. This was continued until a yellow-green color marking became visible on the Pb-VCD. Further, the Lock Advancement Tube was carefully advanced inwards while the Pb-VCD was held in place with steady pressure. When a click was heard, the entry site was considered closed. Extravasally, a hemostatic bovine collagen pad was connected to an intra-arterial portion by a non-absorbable polyester suture. This sandwich was stabilized by a steel clip. After hemostasis, the guide wire was removed. The anchor used has anti-thrombogenic properties.

Anticoagulation and pre-existing antiplatelet therapy were not modified before the TAVR procedure. The administration of new oral anticoagulants (NOACs) was stopped at least 24 h before the TAVR treatment. During the procedure, intravenous unfractionated heparin was used to achieve a target activated clotting time.

### 2.4. Assessment of Haemostasis

To reduce the activated clotting time after TAVR, a reduced dose of protamine (500 IU per 1000 IU of heparin) was administered according to local standard. Here, the target value was less than 250 s. If a residual bleeding was observed after Pb-VCD application, manual compression was performed for at least 3 min. Endovascular measures were applied if further bleeding was observed or if bleeding was substantial. No additional VCD was used. The time from Pb-VCD application until complete hemostasis of the access site was recorded by an independent observer. The adequacy of access site closure was ensured angiographically.

### 2.5. End Point

The primary endpoint of this trial was the rate of major and minor vascular complications, as defined by VARC-3 criteria. Definitions of VARC-3 criteria are outlined in the Supplementary Material. The use of VARC-3 was chosen to ensure application of the most up-to-date, standardized, and clinically relevant endpoint definitions, thereby improving comparability with contemporary studies. Secondary endpoints included VARC-3 bleeding events, length of hospital stay and in-hospital mortality [17]. Bail-out stenting was performed as the preferred and only interventional method for the treatment of vascular complications if conservative methods, including mechanical compression for at least three minutes, was not enough to stop the bleeding.

### 2.6. Statistical Analysis

The data was analyzed using R Software, v.4.5.2. and SPSS^®^ Statistics 29 software (IBM^®^, New York, NY, USA). All tests are two-sided and *p* < 0.05 is considered statistically significant. Tests for normal distribution were applied to metric variables. The Kolmogorov–Smirnov test and the interpretation of the graphical Quantile–Quantile plot were performed. For normally distributed variables, the hypothesis test was carried out using the *t*-test for independent samples. For non-normally distributed variables, the Mann–Whitney U-test was used. If univariate nominal variables were compared with each other, the chi-square test was performed. If the requirements for the chi-square test were not met (<5 expected observations), Fisher’s Exact Test was performed.

A 1:1 nearest neighbor propensity score matching using R Software was applied for imbalanced baseline characteristics including gender, access site CFA diameter, and ilio-femoral tortuosity. The propensity score was estimated using a logistic regression model. Patients were matched applying a caliper width of 0.25 of the standard deviation of the logit of the propensity score, in line with commonly accepted recommendations.

## 3. Results

### 3.1. Baseline Characteristics

A total of 293 patients treated via TF-TAVR were included in propensity score matching, resulting in 85 patient pairs. All patients received the MANTA (Teleflex Inc.^®^, Morrisville, NC, USA) Pb-VCD for closure of the large bore access site; 14F or 18F diameter devices were used. Before propensity score matching was performed, the mean age of the population was 82.2 ± 5.6 years and 68.3% vs. 33.6% (*p* < 0.01) were female patients. Low rates of peripheral vascular disease (10.4% vs. 10.0%, *p* = 0.92) and intermediate surgical risks (EuroSCORE II: 5.8% ± 4.8% vs. 6.4% ± 4.4%, *p* = 0.84) were assessed. Table 1 displays the baseline data of the 14F- and 18F-VCD groups before and after propensity score matching. Differences in the groups before matching included gender (68.3% females (Sb-VCD) versus (vs.) 33.6% (Pb-VCD), *p* < 0.01), access site CFA diameter (7.9 ± 1.5 vs. 8.8 ± 1.5, *p* < 0.01), and ilio-femoral tortuosity (18.0% vs. 38.2%, *p* < 0.01).

### 3.2. Propensity Score Matching and Procedural Data

**Figure 1:**
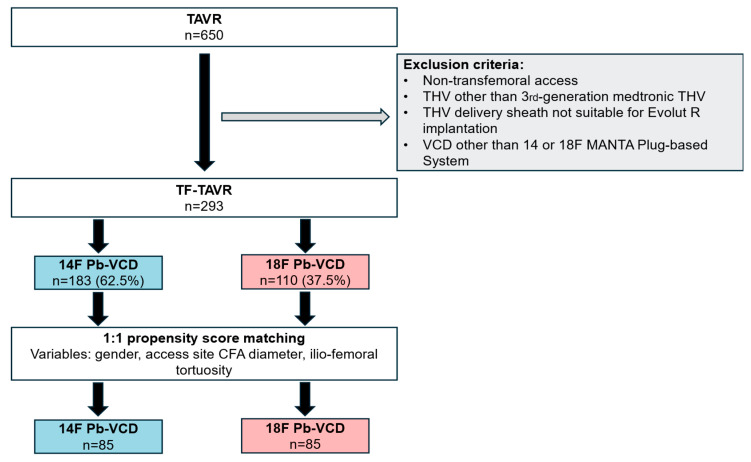
Study flowchart illustration inclusion and exclusion criteria for propensity score matching. CFA, common femoral artery; TAVR, transcatheter aortic valve replacement; THV, transcatheter heart valve; TF, transfemoral; VCD, vascular closure device; Pb-VCD, plug-based vascular closure device.

Propensity score matching was performed for variables with significant differences at baseline (Figure 1), resulting in 85 patient pairs. The only sheath type used was Medtronic Sentrant^®^. For the intraprocedural implantation of the new aortic valve, sizes between 23 mm up to 34 mm were used (23, 26, 29, 34 mm). All of them were third-generation heart valves and devices sized 34 mm were only implanted via the 18F sheath system with the subsequent use of an 18F Pb-VCD. Intravenous protamine was administered before large-bore sheath removal in all patients. No additional VCD was used to achieve hemostasis. Time to vascular closure was equally distributed between the groups (5 (4–8) minutes vs. (5 (4–8) minutes, *p* = 1.00). There was no difference in the duration of the procedure (50 (45–63) minutes vs. 55 (45–70), *p* = 0.14), but fluoroscopy time was statistically higher in the 18F group (9 (7–12) vs. 10 (8–15) min; *p* = 0.02).

### 3.3. In-Hospital Outcome

**Figure 2:**
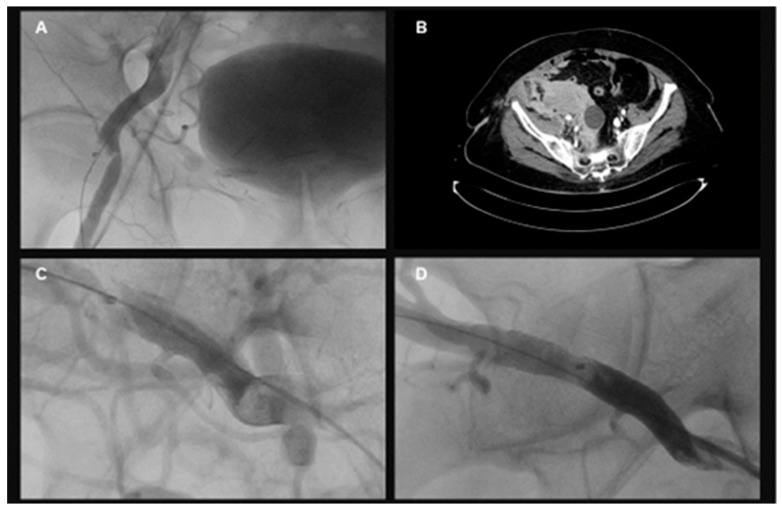
Exemplary imaging data (**A**–**D**) showing (**A**) stenosis of the common femoral artery (CFA) after 14 French (F) plug-based vascular closure device (Pb-VCD) use during transcatheter aortic valve replacement (TAVR), (**B**) persistent bleeding out of the CFA following 14F Pb-VCD use during TAVR. Source of the bleeding is the puncture site for transfemoral TAVR with a retroperitoneal bleeding and hematoma formation, (**C**) dissection of the CFA after 14F Pb-VCD during TAVR and (**D**) interventional treatment of the vascular complication seen under ‘C’ via stenting of the CFA after 14F Pb-VCD use during TAVR.

**Figure 3:**
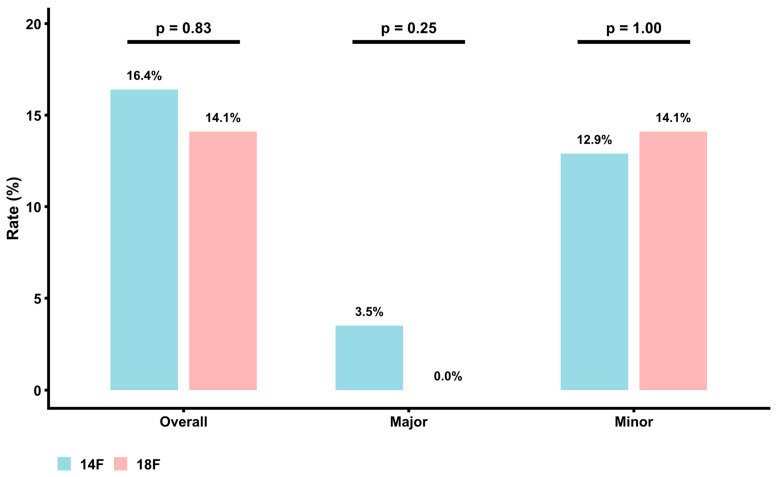
Overall, major and minor valve academic research consortium-3 (VARC-3)-defined vascular complications after 14 or 18 French (F) plug-based vascular closure device (Pb-VCD) use.

The primary endpoint consisting of major and minor vascular complications according to the VARC-3 criteria was balanced between both groups. Exemplary angiographic images of vascular complications and their treatment are shown in Figure 2. Major VARC-3-defined vascular complications occurred in 3.5% of the 14F Pb-VCD group and in 0.0% of the 18F Pb-VCD group (*p* = 0.25). Minor VARC-3-based complications were more frequently observed (12.9% (14F Pb-VCD) versus (vs.) 14.1% (18F Pb-VCD), *p* = 1.00), but without statistical significance (Figure 3). Secondary endpoints including VARC-3-defined bleeding events (*p* = 1.00), length of hospital stay (*p* = 0.34), and in-hospital mortality (*p* = 1.00) were also equally distributed. Details regarding the in-hospital outcome are provided in Table 2.

## 4. Discussion

The present study evaluated the safety of 14 F Pb-VCD for percutaneous vascular access site closure in patients undergoing TF-TAVR. We aimed to evaluate the incidence of VARC-3-defined vascular complications in patients treated with 14F Pb-VCDs and to introduce initial data comparing small 14F Pb-VCDs to larger 18F devices for percutaneous access site closure during TAVR. Comparing outcomes between patients treated with 14F versus 18F Pb-VCD provided insight into whether escalation to a larger Pb-VCD is associated with higher complication rates. The main findings are: (i) 14F Pb-VCDs are associated with overall high rates of vascular complications; (ii) in comparison to larger 18F devices, smaller 14F Pb-VCDs had numerical higher rates of major and comparable incidences of minor vascular complications; (iii) VARC-3 bleeding events were the most common type of vascular complications observed; and (iv) adequate vascular closure following TF-TAVR remains a problem of high clinical significance and is challenged by heterogenous vascular phenotypes.

The MANTA (Teleflex Inc.^®^, Morrisville, NC, USA) Pb-VCD is a well-established and safe alternative to Sb-VCD for access site closure after large bore arteriotomies [10]. Data evaluating safety of the 14F Pb-VCD following TF-TAVR is scarce and conflicting. Recently, the evaluation of vascular access site complications in Pb-VCDs only focused on 18F devices [5,18]. From a clinical perspective, understanding outcome differences between these two commonly used closure device sizes is relevant for procedural planning, risk stratification, and post-procedural surveillance. In our study, we aimed to evaluate the safety of a small 14F Pb-VCD for percutaneous access site closure during TAVR.

Previously, 18F Pb-VCDs were associated with vascular complications in 10.7–19.4% of patients [5,18] and all-cause mortality rates ranged between 0 and 2.7% [5,7,19]. Prospective randomized trials showed the highest rates of complications with 19.4% for the 18F device [5]. In contrast, early studies showed significantly fewer adverse events, with only 3.7% [20]. Only limited data regarding the efficacy and safety of 14F Pb-VCDs during TAVR exists. Recently, our group and others showed increased access site complications driven primarily by minor complications in Pb- compared to Sb-VCD [21,22]. However, most other prior data available was reported during early MANTA (Teleflex Inc.^®^, Morrisville, NC, USA) days with only cumulative evaluation of 14F and 18F devices together, low numbers of included patients or evaluation of complications according to outdated VARC-2 criteria [10,11,23]. Further, heterogenous definitions of endpoints exist in already published studies including VARC-2 or -3 criteria or, by contrast, only non-specified rates of device failure [5,10,24]. In our study, currently recommended VARC-3 criteria regarding vascular and access-related complications were used to define the primary endpoint and to ensure that a widely used system for the assessment of vascular complications is in place [17].

The population analyzed in this study was treated at a high-volume, experienced center where vascular access closure is consistently performed using Pb-VCDs. Both 14F and 18F Pb-VCDs were applied in a substantial number of patients, allowing for a balanced comparison. The primary endpoint, defined as VARC-3 major and minor vascular complications, was comparable between the 14F and 18F groups. Likewise, no significant differences were observed in bleeding events or arterial obstruction due to occlusion. Overall mortality rates were low and similar between groups. These findings suggest that, despite the smaller device profile, the use of 14F Pb-VCDs does not translate into a measurable reduction in vascular complication rates compared with 18F systems in this real-world cohort. Accordingly, device selection in clinical practice may be guided more by procedural requirements and vascular anatomy than by expectations of differential complication risk alone.

Our findings do not necessarily contradict previous findings and suggested higher complication rates of 18F Pb-VCD in randomized trials but likely reflect differences in study context and methodology. Our analysis uses VARC-3 definitions, whereas earlier studies may have applied different endpoint frameworks, had varying center experience, learning curves and evolving access strategies (e.g., puncture optimization, bail-out protocols).

As stated by the manufacturer (MANTA, Teleflex Inc.^®^, Morrisville, NC, USA), 18F Pb-VCDs are contraindicated in vessels sized less than 6 mm and 14F Pb-VCDs in vessels sized less than 5 mm with further recommendation for avoidance in anatomies with posterior calcifications. This indicates that our study population is at a low-to-moderate risk for vascular complications given the rates of vascular calcifications and the large vessel diameter. Regarding this interpretation, rates of VARC-3 vascular complications are unexpectedly high. Since data on the safety and feasibility of 14F Pb-VCDs is limited, our work could lay the groundwork for further studies dedicated to the investigation of the efficacy and safety of 14F Pb-VCDs. Our results are likely generalizable to other centers with comparable expertise and procedural volume, although multicenter studies would be required to confirm broader applicability.

In summary, Pb-VCDs cause vascular complications irrespective of the device size. This could be related to the ease of use and by the patient’s anatomy more than the vascular closure technology. On the other hand, this propensity-matched study showed lower rates of VARC-3-defined major vascular complications compared to previously published studies. These findings require further confirmation using future randomized trials. Here, different closure devices could be compared in settings with different baseline complexities. Since numbers of transfemoral catheter procedures such as TAVR or endovascular aortic repair are steadily increasing, it is important to identify the optimal vascular closing technique for reliable access site closure and to ensure that already available VCDs are of high quality in different sizes. Various vascular phenotypes in age-impacted patient cohorts offer complex clinical scenarios in which the evaluation of the safety and efficacy of 14F Pb-VCDs used during TAVR could contribute to reduced morbidity and mortality. 

## 5. Limitations

No ultrasound evaluation of the access site was performed, which absence may have influenced absolute complication rates. In addition, a more detailed assessment of included patients in terms of follow-up imaging, including ultrasonic evaluation of femoral patency, would be desirable. We acknowledge that the single-center design and high operator experience may limit the generalizability of the results to lower-volume centers or operators earlier in their learning curve. Due to the retrospective single-center design, selection bias cannot be fully excluded. However, consecutive inclusion of all eligible patients without additional selective exclusion was intended to minimize this risk. All patients were treated by experienced interventional cardiologists with consistent rates of complications across operators; thus, a difference in treatment and intervention strategy as well as an impact of learning curve can be excluded. A formal a priori power calculation was not performed due to the retrospective nature of the study; therefore, the findings should be interpreted with caution despite the consistent and clinically relevant effect sizes observed. Despite propensity score matching and well-balanced baseline characteristics, residual confounding cannot be fully excluded due to the non-randomized design and the potential influence of unmeasured anatomical and procedural factors.

## 6. Conclusions

There is no significant difference in the incidence of VARC-3-defined major or minor vascular complications between 14 and 18F Pb-VCD groups in our study. However, we present the first data on 14F Pb-VCDs compared to standard 18F devices, which could lay the groundwork for further prospective trials and comparisons between 14F Pb-VCDs and 14F suture-based closure techniques. 

## Figures and Tables

**Table 1:** Baseline characteristics.

n	*Entire cohort* 14F-VCD 183	18F-VCD 110	*p* Value	*Matched cohort* 14F-VCD 85	18F-VCD 85	*p* Value
Age (years)	82.1 ± 5.7	82.3 ± 5.2	0.75	83 (79.6–83.1)	81 (78.9–81.9)	0.99
Females, n (%)	125 (68.3)	37 (33.6)	**<0.01**	36 (42.4)	36 (42.4)	1.00
Body mass index, (kg/m^2^)	27.1 ± 5.1	27.7 ± 5.7	0.42	26.6 (24.6–30.1)	26.5 (24.0–30.0)	0.92
EuroSCORE 2	6.1 ± 6.1	6.4 ± 5.2	0.84	4.5 (3.0–6.7)	4.5 (3.3–7.6)	0.58
Coronary artery disease, n (%)	97 (53.0)	65 (59.1)	0.31	52 (61.2)	50 (58.8)	0.75
Coronary artery bypass grafting, n (%)	10 (5.5)	11 (10.0)	0.21	4 (4.7)	8 (9.4)	0.37
Percutaneous coronary intervention, n (%)	56 (30.6)	36 (32.7)	0.73	31 (36.5)	29 (34.1)	0.563
Peripheral artery disease, n (%)	19 (10.4)	11 (10.0)	0.92	7 (8.2)	8 (9.4)	0.78
Atrial fibrillation, n (%)	67 (36.6)	37 (33.6)	0.61	27 (31.8)	27 (31.8)	1.00
COPD	14 (7.7)	5 (4.6)	0.30	6 (7.1)	4 (4.7)	0.75
Previous stroke, n (%)	16 (8.7)	9 (8.2)	0.87	11 (12.9)	5 (5.9)	0.12
Diabetes, n (%)	15 (8.2)	11 (10.0)	0.59	8 (9.4)	8 (9.4)	1.00
GFR (mL/min)	52.1 ± 18.3	55.0 ± 19.1	0.20	54 ± 18	55 ± 19	0.85
Anemia (Hb < 11 g/dL), n (%)	70 (38.3)	37 (33.6)	0.43	28 (32.9)	30 (35.3)	0.75
Access site CFA diameter (mm)	7.9 ± 1.5	8.8 ± 1.5	**<0.01**	8 (7–9)	8 (7–9)	0.45
Ilio-femoral tortuosity, n (%)	33 (18.0%)	42 (38.2%)	**<0.01**	20 (23.5)	20 (23.5)	1.00
CFA calcification ≥moderate, n (%)	39 (21.3%)	23 (20.1%)	0.94	20 (23.5)	25 (29.4)	0.38
LVEF (<35%), n (%)	15 (8.2)	16 (14.6)	0.09	6 (7.1)	12 (14.1)	0.14

Note: Variables highlighted in bold are significantly different between the groups. Abbreviations: Hb, hemoglobin; CFA, common femoral artery; COPD, chronic obstructive pulmonary disease; GFR, glomerular filtration rate; LVEF, left ventricular ejection fraction.

**Table 2:** Procedural characteristics and outcome after transcatheter aortic valve replacement with 14 and 18 French plug-based vascular closure systems (Pb-VCD).

	14F-VCD	18F-VCD	*p* Value
Patients, n	85	85	
*Procedural data*			
THV, n (%)			
Evolut R/PRO©	85 (100)	85 (100)	1.00
THV size (mm), n (%)			
23	2 (2.4)	0 (0.0)	0.50
26	22 (25.9)	20 (23.5)	0.74
29	61 (71.8)	32 (37.6)	**<0.01**
34	0 (0.0)	33 (38.8)	**<0.01**
Procedural sheet, n (%)			
Medtronic Sentrant	85 (100)	85 (100)	1.00
Fluoroscopy time (min)	9 (7–12)	10 (8–15)	**0.02**
Contrast agent (mL)	200 (155–250)	200 (150–150)	0.75
Procedural duration (min)	50 (45–63)	55 (45–70)	0.14
Time to vascular closure (min)	5 (4–8)	5 (4–8)	1.00
*Outcome*			
Overall vascular complication, n (%)	14 (16.4)	12 (14.1)	0.83
Major vascular complication, n (%)	3 (3.5)	0 (0.0)	0.25
Minor vascular complication, n (%)	11 (12.9)	12 (14.1)	1.00
Bleeding, n (%)	9 (10.6)	8 (9.4)	1.00
*Type 1*	5 (5.9)	6 (7.1)	1.00
*Type 2*	1 (1.2)	2 (2.4)	1.00
*Type 3*	2 (2.4)	0 (0.0)	0.50
*Type 4*	1 (1.2)	0 (0.0)	1.00
Vessel stenosis, occlusion, dissection, n (%)	4 (4.8)	4 (4.8)	1.00
Length of hospital stay (days)	6 (5–8)	6 (5–7)	0.34
In-hospital CV mortality, n (%)	1 (1.2)	1 (1.2)	1.00

Note: Variables highlighted in bold are significantly different between the groups. Abbreviations: CV, cardiovascular; THV, transcatheter heart valve.

## Data Availability

Data is provided within the manuscript and can further be assessed through contacting the corresponding author upon reasonable request.

## Supplementary Materials

The following supporting information can be downloaded at: https://www.mdpi.com/article/10.3390/jcm15083095/s1.

## Abbreviations

Pb-VCD	plug-based vascular closure device.
Sb-VCD	suture-based vascular closure device.
F	French.
TAVR	transcatheter aortic valve replacement.
AS	aortic stenosis.
SAVR	surgical aortic valve replacement.
CFA	common femoral artery.
VARC-2	valve academic research consortium-2 criteria.
VARC-3	valve academic research consortium-3 criteria.
VCD	vascular closure device.
PLGA	polylactide-co-glycolide.
NOAC	new oral anticoagulants
