# Supplementary material for: Vascular Complications in Transcatheter Aortic Valve Replacement Using 14 vs. 18 French Plug-Based Percutaneous Closure Devices: A Propensity Score-Matched Observational Study
Source: J Clin Med. 2026 Apr 18;15(8):3095. doi: 10.3390/jcm15083095 (PMC13116924; doi:10.3390/jcm15083095)
Supplement: Supplementary file 1 [file jcm-15-03095-s001.zip › jcm-4236464-supplementary.pdf]

Online Supplementary Material

Vascular complications in transcatheter aortic valve replacement using 14- vs 18-French plug-based percutaneous closure devices: a propensity score-matched observational study

*Tobias Lerchner[1,] Norvydas Zapustas[2], Melchior Seyfarth[1, 2], Klaus Tiroch[3], David Holzhey[1,4], * Marc Vorpahl[1,5]*

[1]Witten-Herdecke University, Germany; [2]Department of Cardiology, Helios University Heart Center Wuppertal, Germany; [3]Department of Cardiology, Heart Center Bodensee, Germany; [4]Department of Cardiothoracic Surgery, Helios University Heart Center Wuppertal, Germany; [5]Department of Cardiology, Helios Heart Center Siegburg, Germany

Corresponding Author:

Prof. Dr. Marc Michael Vorpahl
Helios Heart Center Siegburg
Ringstrasse 49
53721 Siegburg, Germany
E-Mail: marc.vorpahl@helios-gesundheit.de

Online supplementary material

VARC-3 criteria included and applied:

Major

- Aortic dissection or aortic rupture.
- Vascular injury (perforation, rupture, dissection, stenosis, ischaemia, arterial or venous thrombosis including pulmonary embolism, arteriovenous fistula, pseudoaneurysm, haematoma, retroperitoneal haematoma, infection) or compartment syndrome resulting in death, limb or visceral ischaemia, or irreversible neurologic impairment.
- Distal embolization (non-cerebral) from vascular source resulting in death, amputation, limb or visceral ischaemia, or irreversible end-organ damage.
- Unplanned endovascular or surgical intervention resulting in death, limb or visceral ischaemia, or irreversible neurologic impairment.
- Closure device failure resulting in death, limb or visceral ischaemia, or irreversible neurologic impairment.

Minor

- Vascular injury not resulting in death, limb or visceral ischaemia, or irreversible neurologic impairment.
- Distal embolization treated with embolectomy and/or thrombectomy, not resulting in death, amputation, limb or visceral ischaemia, or irreversible end-organ damage.
- Any unplanned endovascular or surgical intervention, ultra-sound guided compression, or thrombin injection, not resulting in death, limb or visceral ischaemia, or irreversible neurologic impairment.
- Closure device failure not resulting in death, limb or visceral ischaemia, or irreversible neurologic impairment.
